# Developing New Treatments for COVID-19 through Dual-Action Antiviral/Anti-Inflammatory Small Molecules and Physiologically Based Pharmacokinetic Modeling

**DOI:** 10.3390/ijms23148006

**Published:** 2022-07-20

**Authors:** Panagiotis Zagaliotis, Anthi Petrou, George A. Mystridis, Athina Geronikaki, Ioannis S. Vizirianakis, Thomas J. Walsh

**Affiliations:** 1 Transplantation-Oncology Infectious Diseases, Weill Cornell Medicine, New York, NY 10065, USA; panagiotis_zag@yahoo.com (P.Z.); thomaswalshmd@gmail.com (T.J.W.); 2 Department of Medicinal Chemistry, School of Pharmacy, Aristotle University of Thessaloniki, 54124 Thessaloniki, Greece; anthi.petrou.thessaloniki1@gmail.com; 3 Laboratory of Pharmacology, School of Pharmacy, Aristotle University of Thessaloniki, 54124 Thessaloniki, Greece; geormyst@pharm.auth.gr; 4 Department of Life & Health Sciences, School of Sciences and Engineering, University of Nicosia, Nicosia 1700, Cyprus; 5 Center for Innovative Therapeutics and Diagnostics, Richmond, VA 23223, USA

**Keywords:** COVID-19, antiviral agents, dual action, PBPK modeling, molecular docking

## Abstract

Broad-spectrum antiviral agents that are effective against many viruses are difficult to develop, as the key molecules, as well as the biochemical pathways by which they cause infection, differ largely from one virus to another. This was more strongly highlighted by the COVID-19 pandemic, which found health systems all over the world largely unprepared and proved that the existing armamentarium of antiviral agents is not sufficient to address viral threats with pandemic potential. The clinical protocols for the treatment of COVID-19 are currently based on the use of inhibitors of the inflammatory cascade (dexamethasone, baricitinib), or inhibitors of the cytopathic effect of the virus (monoclonal antibodies, molnupiravir or nirmatrelvir/ritonavir), using different agents. There is a critical need for an expanded armamentarium of orally bioavailable small-molecular medicinal agents, including those that possess dual antiviral and anti-inflammatory (AAI) activity that would be readily available for the early treatment of mild to moderate COVID-19 in high-risk patients. A multidisciplinary approach that involves the use of in silico screening tools to identify potential drug targets of an emerging pathogen, as well as in vitro and in vivo models for the determination of a candidate drug’s efficacy and safety, are necessary for the rapid and successful development of antiviral agents with potentially dual AAI activity. Characterization of candidate AAI molecules with physiologically based pharmacokinetics (PBPK) modeling would provide critical data for the accurate dosing of new therapeutic agents against COVID-19. This review analyzes the dual mechanisms of AAI agents with potential anti-SARS-CoV-2 activity and discusses the principles of PBPK modeling as a conceptual guide to develop new pharmacological modalities for the treatment of COVID-19.

## 1. Introduction

In December 2019, several cases of pneumonia of unknown etiology were reported in the city of Wuhan in China [1]. In January 2019, the cause of these pneumonia cases was identified to be a novel coronavirus, which was given the name “Severe Acute Respiratory Syndrome Coronavirus 2” (SARS-CoV-2) by the International Committee on the Taxonomy of Viruses [2]. The disease caused by SARS-CoV-2 was named “coronavirus disease 2019” (COVID-19) by the World Health Organization (WHO). As of today, COVID-19 has approximately half a billion cases and has resulted in more than six million deaths worldwide, according to the WHO [3].

There is a dearth of small-molecule medicines for the treatment of symptomatic COVID-19. Two classes of small molecules are currently utilized: inhibitors of the inflammatory cascade, such as dexamethasone and baracitinib, and inhibitors of the cytopathic effect of the virus, such as molnupiravir or nirmatrelvir/ritonavir [4]. There is a critical need for an expanded armamentarium of orally bioavailable medicines, including those that possess dual antiviral and anti-inflammatory (AAI) activity that would be available for the early treatment of mild to moderate COVID-19 in high-risk patients [5,6]. This paper will review the mechanisms of the dual AAI activity of antiviral agents against SARS-CoV-2 and further discuss the role of physiologically based pharmacokinetic (PBPK) modeling in developing new medicines for the treatment of COVID-19.

Physiologically based pharmacokinetic (PBPK) modeling is a mathematical method for modeling the absorption, distribution, metabolism, and elimination of medicinal compounds in animals and humans. PBPK involves the multidisciplinary approach use of in silico screening tools to identify the potential drug targets of an emerging pathogen, as well as in vitro and in vivo models for the determination of a drug’s efficacy and safety [7]. Characterization of candidate AAI molecules with PBPK modeling provides critical data for accurate dosing. This review therefore analyzes the dual mechanisms of AAI agents with potential anti-SARS-CoV-2 activity and discusses the principles of PBPK modeling as a conceptual guide to new therapeutic options for the treatment of COVID-19.

### 1.1. Molecular Targets of SARS-CoV-2

SARS-CoV-2 is an enveloped, positive-sense, single-stranded RNA betacoronavirus of the family Coronaviridae [2]. The viral genome encodes a total of 14 open reading frames (ORFs), which encode 29 proteins [8]. Four of these proteins are structural proteins: the spike (S), envelope (E), membrane (M) and nucleocapsid (N) proteins. The rest are non-structural proteins (NSPs), of which 15 are encoded as a large polyprotein (pp1ab) by the first ORF, and the rest are encoded by several ORFs. The pp1ab polyprotein is then cleaved by the NSP-5 (or main protease) into distinct proteins. Even though, in principle, all proteins of SARS-CoV-2 are potential drug targets, the most attractive ones are the S protein, on which the virus relies to enter the cells [9], and the main protease, which dictates the life cycle of the virus by directly affecting the production of most other proteins of the virus [10]. Other proteins of SARS-CoV-2 have also been used as potential drug targets [11], with the most prominent example being the RNA-dependent RNA polymerase (RdRp), which is targeted by the approved drug remdesivir [12] (Table 1).

### 1.2. Targets for Anti-Inflammatory Agents against COVID-19

Manifested initially by cough, dyspnea, fever, and hypoxemia, COVID-19 may progress to a cytokine storm, lethal respiratory disease, and multi-organ failure. COVID-19 can be divided into three phases: (i) an asymptomatic phase with or without detectable virus, (ii) a mild symptomatic phase with upper airway involvement and manifestations, and (iii) a severe, acute phase with high viral role and respiratory distress, hypoxia, and progression to acute respiratory distress syndrome (ARDS) [13]. ARDS is believed to be the main reason for death in COVID-19, and it is characterized by the uncontrolled release of pro-inflammatory cytokines and chemokines, known as the “cytokine storm” [14], which results in lung damage and multiple organ failure. The mechanisms involved in the cytokine storm have been reported in many articles [15,16,17] based on studies on SARS-CoV and MERS-CoV, as well as SARS-CoV-2. More specifically, the open reading frames (ORFs) [18] of the virus genome activate immune pathways, which results in the upregulation of pro-inflammatory cytokines, such as IL-1, IL-2, IL-6, IL8, IL-17, G-CSF, and GM-CSF, and chemokines such as IP-10 and MCP-1, while suppressing antiviral mechanisms, such as type-I interferon (IFN). For example, ORF7a activates nuclear factor-κB (NF-κB) and ORF6 limits interferon production, while ORF3a induces necrotic cell death [19]. Another important aspect responsible for complications and eventually death in critically ill patients is the development of pulmonary thromboembolism, which further contributes to hypoxia and lung injury. Cyclocyxenase-2 (COX-2) and 5-lipoxygenase (5-LOX), among others, are important mediators of the cytokine storm, which intensify and worsen tissue morbidity related to COVID-19 [20,21]. Inhibition of these inflammatory pathways by agents such as dexamethasone and baricitinib has significantly improved outcomes in patients with COVID-19.

### 1.3. Antiviral Agents with No Significant Anti-Inflammatory Activity

#### 1.3.1. Remdesivir

Remdesivir is a nucleoside analog that acts as a competitive inhibitor of RNA-dependent RNA polymerase (RdRp) and has previously been used against Ebola, as well as being tested against MERS-CoV, SARS-CoV, and other coronaviruses. In 2020, remdesivir was added to the “Solidarity” international clinical trial, which began on 18 March 2020, developed by the World Health Organization (WHO) in an effort to produce a timely response to the emerging COVID-19 threat [22]. Remdesivir has produced less-than-optimal results, but has, however, been approved by the FDA for use against COVID-19, as it was deemed the best of the available drugs against the disease at the time [12].

In terms of PBPK profiling, Luts et al., created models that accurately predict observed adult PK profiles of remdesivir to predict pediatric PK profiles and metabolites’ steady-state exposure. They use the Pediatric Population Model of the Simcyp simulator, incorporating relevant physiologic and mechanistic information. Their model supported the currently used dosing regimens in pediatric clinical trials and the emergency use authorization and pediatric compassionate use programs [23].

In his work James Gallo created a hybrid PBPK model with each tissue presented as a two-compartment model (extracellular and intracellular component) to predict remdesivir’s active triphosphate nucleoside (TN) metabolite concentration in different tissues. He found that current clinical dosing regimens are successful at achieving the desired TN concentrations [24].

In another in silico work, Deb and Reeves, using Gastroplus© software, simulated the physiological properties of remdesivir using its predictive modules (ADMET predictor and PKPlus), as well as the PK profiles of remdesivir, examining its behavior for DDIs as well as its properties in special populations that are expected to be especially affected by SARS-CoV-2 (based on age, weight, liver function, and renal function status). In the case of DDIs, they checked against other SARS-CoV-2 medications as well as drugs that are commonly used for comorbidities. They concluded that GS-5734, an inactive prodrug, shows superiority compared to remdesivir, as the latter’s disposition can be affected by physiological and pathological conditions and DDIs. Remdesivir has no significant immunomodulatory activity [25].

#### 1.3.2. Lopinavir–Ritonavir

Lopinavir is an inhibitor of the HIV-1 protease [26], and it has also shown in vitro activity against SARS-CoV-2, SARS-CoV, and MERS-CoV [27,28]. Ritonavir is also an HIV protease inhibitor, and is usually administered in combination with lopinavir, to increase the plasma half-life of the latter. Similar to remdesivir, lopinavir–ritonavir has no significant immunomodulatory activity. Using approaches of PBPK modeling, Niu et al., built models for Caucasian and Chinese populations and, using simulated free plasma and lung concentration values, they performed PK/PD correlations. They validated their model against several clinical datasets and found that the current lopinavir–ritonavir regime of 400/100 mg BID fails to achieve adequate lung concentrations and, although higher concentrations are achieved both in plasma and in the lungs in the Chinese population, a significant dose increase is necessary to reach the EC_50_ value for both populations. They concluded, however, that the increase in dosage could increase potential side effects (e.g., QT prolongation), thus suggesting to carefully weigh these concerns before administration [29].

Another possible drug combination involving ritonavir is its combination with nifedipine, a calcium channel blocker, since hypertension is considered a significant co-morbidity in COVID-19 patients. However, as ritonavir is a strong CYP3A4 inhibitor, it is possible that a DDI between ritonavir and nifedipine occurs. Indeed, by developing and validating models for both drugs using PBPK modeling approaches, the researchers found a strong interaction between the two drugs that could potentially lead to severe hypotension (>40 mg Hg drop in blood pressure), recommending against the concomitant use of these two drugs [30].

#### 1.3.3. Molnupiravir

Initially used against influenza viruses and alpha encephalitis viruses, molnupiravir possesses an interesting mechanism of action to exert its antiviral activity. More specifically, this molecule uses the mechanism of “error catastrophe”, by increasing the rate of mutation of the viral genome beyond a certain threshold. This makes viral survival impossible, since the virus cannot tolerate such a high degree of mutation [31].

In more detail, molnupiravir is cleaved in plasma by host esterases to an active nucleoside analog, β-D-N4-hydroxycytidine (NHC) or EIDD-1931 [32]. This active form of the drug is distributed to various tissues and subsequently converted to its corresponding 5′-triphosphate (NHC triphosphate or MTP). This then targets the RdRp, which is virally encoded and competitively inhibits the cytidine and uridine triphosphates and incorporates M instead. The RdRp uses the NHC triphosphate as a substrate instead of the cytidine and uridine triphosphates and then incorporates either A or G in the RdRp active centers, forming stable complexes and thus escaping proof reading by the synthesis of a mutated RNA [33].

Molnupiravir also resulted in a reduction in inflammatory biomarkers in an in vivo study in a ferret model of influenza infection [34], but it has not shown direct anti-inflammatory activity in SARS-CoV-2 infections, which would render it a dual-activity compound.

Despite its significant advantages against SARS-CoV-2, the mechanism of action of molnupiravir entails the risk of causing mutations to the human genome, and therefore inducing side effects to the host, or potentially leading to the appearance of new SARS-CoV-2 variants [35]. However, it is believed that the proposed duration of treatment is so short that there is not sufficient time for these side effects to manifest.

#### 1.3.4. Nirmatrelvir–Ritonavir

Nirmatrelvir–ritonavir is another orally bioavailable molecule that has been added to the armamentarium of compounds with anti-SARS-CoV-2 activity. Its mechanism of action entails the inhibition of the main protease of SARS-CoV-2. Nirmatrelvir came about after the modification of a molecule called PF-07321332, which had been investigated against SARS-CoV [36]. PF-07321332 was abandoned due to its low passive absorptive permeability and poor oral absorption. To improve these properties, a new compound, nirmaltrelvir, was created with a reversible covalent thioimidate adduct [37]. Nirmaltrelvir exhibited higher oral absorption and was therefore deemed a good candidate for an anti-SARS-CoV-2 compound.

In more detail, nirmaltrelvir inhibits the main protease of SARS-CoV-2 by binding to its active site through S–C bonds. This complex is further stabilized through hydrogen bonds and hydrophobic interactions [37]. This binding takes place in two steps: an initial non-covalent addition with the dyad in a neutral form with the formation of the thiolate–imidazolium ion pair, and ligand relocation for finalizing the electrophilic attack [37].

### 1.4. Dual-Activity Compounds as Potential Drug Candidates against COVID-19

Notably, most existing drug regimens against COVID-19 involve the use of multiple compounds. This is likely attributed to two reasons: first, that the use of a single agent that only targets one key pathway of the disease has not yet been shown to be effective in improving clinical outcomes, and second, that no agent has yet been shown to address two or more aspects of the disease simultaneously, (i.e., the cytopathic effect of the virus and the inflammatory cascade that derives from it). Considering the potential drawback of a more-than-one-compound approach, which is the potential drug interactions or the metabolic burden likely to be placed on the patients from the many drugs, it is interesting that the possibility of dual-activity compounds, which would eliminate these considerations, has been so far overlooked.

Many of the compounds that are already being used or are being evaluated as potential anti-SARS-CoV-2 compounds potentially have multiple protein targets to tackle the proliferation of the virus. However, few, if any, of these possess dual inhibitory activity in the sense of both antiviral and anti-inflammatory action. The use of more than one drug in the treatment protocols of COVID-19, of which some exert antiviral properties and some anti-inflammatory action, showcases the need for molecules that possess a broader spectrum of activity, in order to address both these key components of the disease, rather than targeting multiple targets within either the viral or the inflammatory component.

Some study groups have described the use of dual antiviral/anti-inflammatory activity compounds for the treatment of COVID-19. Of these compounds, some are in the developmental stage, whereas others are already in clinical trials.

#### 1.4.1. Nitazoxanide

In another case of repurposing an antimicrobial drug, Rajoli et al. [38] simulated, using PBPK modeling, the PK profiles of nitazoxanide, an anthelmintic drug that has shown in vitro activity against SARS-CoV-2 [38]. Nitazoxanide owes its anti-inflammatory properties to its inhibition of the production of several pro-inflammatory cytokines, including IL-6, IL-8, and TNF-α, in peripheral blood mononuclear cells. It was also shown, in an in vivo murine model, to decrease the IL-6 production induced by thioglycolate and lipopolysaccharide [39]. The researchers were able to predict optimal dosing schemes, thus providing a rational basis for the design of clinical trials for this drug [38].

#### 1.4.2. Chloroquine, Hydroxychloroquine, and Azithromycin

Hydroxychloroquine is an aminoquinoline used for the treatment of malaria. Its proposed mechanism of action for COVID-19 entails the inhibition of Toll-like receptors (TLRs), which participate in the upregulation of pro-inflammatory cytokines, resulting in the lung and other tissue damage involved in COVID-19 [40]. However, this agent has not managed to significantly reduce mortality in COVID-19 patients [41].

Azithromycin is a macrolide antibiotic, used in patients with COVID-19 on the basis of its antimicrobial properties for the prevention of secondary bacterial infections that might arise, as well as its antiviral properties with potential activity against SARS-CoV-2 [42].

Despite their potential use against COVID-19 based on their mechanism of action, these molecules are highly affected by changes in the pH of the lungs due to infections, resulting in altered PK profiles of these drugs in the lungs. Using PBPK modeling approaches and a mechanistic lung model, Yeo et al., simulated the PK profiles of chloroquine, hydroxychloroquine, and azithromycin. They found that changes (reduction) in the pH of the lungs can lead to increased exposure of the drugs locally, with a minimal overall change in the plasma concentration profiles. They also found that if renal impairment is a comorbidity, the increase in local exposure is even greater. Their work aimed at presenting the capabilities of such approaches, rather than informing pharmacotherapy [42].

The Dutch Centre for Infectious Disease Control has included chloroquine against SARS-CoV-2 disease. This, and the fact that the World Health Organization dosing guidelines for children have been found to be suboptimal, led Verscheijden et al., to calculate the optimal pediatric dose for different ages utilizing PBPK modeling approaches [43]. Specifically, they calculated the optimal dosage per kg for different pediatric ages to achieve the PK profile of adults. They support the notion of age-adjusted dosing, thus avoiding either suboptimal or toxic drug levels in children.

Furthermore, in their work, Cui et al., used PBPK modeling to create models to predict the concentration profiles of chloroquine in different tissues. They did so by building a PBPK model using drug data extrapolated from animal sources. This way, they proposed optimized dosage regimens for patient with SARS-CoV-2 infection [44]. Finally, Zhang et al., created a PBPK model for hydroxychloroquine to support dosing design. They focused on drug absorption and disposition mechanisms to support optimal dosing in specific populations that take concomitant medications, young children, the elderly, subjects with organ impairment, inhibitors, and pregnant women. They built the model using in vitro and in vivo experiments, validated it using published references, and informed its parameters through experiments in monkeys. They predicted the HCQ distribution in lung tissues using a permeability-limited lung model. Their aim was to better inform dosing for clinical trials [45].

Following a similar principle, several other small molecules that were previously being used for the treatment of other viral infections were tested against SARS-CoV-2 infection. Additionally, the use of non-small-molecule agents has been reported and is applied in clinical practice, such as monoclonal antibodies or the plasma of patients who have recovered from COVID-19 [46].

#### 1.4.3. Sabizabulin (Veru-111)

Sabizabulin is an orally bioavailable drug in the developmental stages against prostate cancer. Its mechanism of action entails targeting, binding to, and crosslinking the alpha and beta tubulin subunits of microtubules and intermediate filaments of cells, resulting in disruption of the cytoskeleton. Furthermore, Veru-111 causes apoptosis, or cell death, by cleaving poly ADP ribose polymerase (PARP), which is important for DNA repair in cancer cells.

Owing to its mechanism of action, it was deemed an attractive alternative for the treatment of COVID-19, being expected to dampen the cytokine storm observed in patients, as well as target the viral cytopathic pathway. In vitro studies further confirmed this hypothesis, where it substantially reduced key cytokines of ARDS. Furthermore, a multi-center, randomized, placebo control clinical trial (NCT04388826) recently presented data according to which sabizabulin has both dual anti-inflammatory and antiviral activity, and it reduced mortality in hospitalized patients with moderate to severe COVID-19.

#### 1.4.4. Opaganib

Opaganib is another small molecule currently in phase II/III clinical trials (NCT04467840), displaying both antiviral and anti-inflammatory activity. More specifically, it is a novel, orally administered, sphingosine kinase-2 (SK2) selective inhibitor. The compound has shown promising preclinical evidence of inhibiting SARS-CoV-2, by inhibiting viral replication, reducing the hyper-immune inflammatory response, and diminishing ARDS-related thrombosis (blood clots). Opaganib has successfully undergone a randomized, double-blind, placebo-controlled phase II clinical trial (NCT04414618).

#### 1.4.5. Selinexor

Selinexor is the first selective inhibitor of nuclear export (SINE) that has been approved for human usage by the FDA [47]. It is currently licensed with dexamethasone for the treatment of patients with refractory multiple myeloma. Selinexor also is being studied as an investigational agent for the treatment of diffuse large B-cell lymphomas, liposarcomas, endometrial cancer, glioblastoma multiforme, and myelodysplasia [47]. It displays potent antiviral activity against a wide range of RNA and DNA viruses, including influenza, Hepatitis C, Venezuelan equine encephalitis virus, HIV, HPV 11, Kaposi’s sarcoma, Herpes virus, Epstein–Barr virus, and Adenovirus. In addition, it possesses anti-inflammatory activity by blocking the expression of NF-κB-mediated cytokines, including TNFα, IL-1β, G-CSF, and IL-6 [48]. Inhibition of XPO1 also blocks the transportation of RXRo export from the nucleus, thereby inhibiting I-O1 beta production and release [48].

Based on its potential dual activity against COVID-19, selinexor was evaluated in an international phase 2b randomized controlled clinical trial of selinexor versus standard of care for the treatment of patients with COVID-19 (Evaluation of Activity and Safety of Oral Selinexor in Participants With Severe COVID-19 Infection (NCT04349098)).

#### 1.4.6. Atazanavir

Atazanavir is a licensed anti-HIV drug, which belongs to the protease inhibitors. More specifically, it binds to the protease active site and inhibits the activity of the enzyme [49]. This inhibition prevents the cleavage of the viral polyproteins, resulting in the formation of immature non-infectious viral particles.

Following molecular docking studies, which showed that atazanavir has a strong binding affinity to the main protease of SARS-CoV-2 [50] and further demonstrating high bioavailability within the respiratory tract [51], atazanavir was deemed an interesting candidate for anti-SARS-CoV-2 activity, and was used in in vivo studies of humanized murine models of SARS-CoV-2 infection [50]. In these studies, it improved survival in atazanavir-treated groups, compared with non-treated controls. In addition, it showed sufficient anti-inflammatory activity, significantly reducing the levels of interleukin-6 (IL-6), tumor necrosis factor-α (TNF-α), and keratinocyte-derived chemokines (KCs) in the lungs of treated mice. Atazanavir further protected animals from severe lung injury, which could lead to hemorrhage and shrinking of the lobe, bronchiole, and alveoli, which was prominent in untreated groups [50].

Based on these promising preclinical data for atazanavir, the drug was registered in phase 2 (*NCT04459286)* and phase 2/3 (*NCT04452565*) clinical trials [52]. The former investigated the efficacy of atazanavir in combination with ritonavir and the standard of care (SOC), in ambulatory patients with confirmed COVID-19 infection by a PCR test. However, the study was closed per the recommendation of the DSMB in February 2022. The latter is investigating the efficacy of atazanavir, alone or with dexamethasone, in comparison with traneurocin (NA-831), a neuroprotective drug, normally used for the treatment of Alzheimer’s disease. The study will further investigate the potential synergy between atazanavir and dexamethasone.

Berlin et al., by combining in vitro tests that examined the pre-absorptive behavior of atazanavir with literature data relating to its permeability and post-absorptive parameters, created physiologically based models to analyze, using sensitivity analysis, the factors that contribute to the oral absorption of atazanavir [53]. They concluded that, for atazanavir, the post-absorptive factor had a more significant role in its absorption and that further optimization in formulation would not induce significant improvements.

In their work, Sychterz et al., used PBPK modeling to examine the UGT1A1-related DDI risk with raltegravir and atazanavir in pregnant populations [54]. They found that the induction of UGT1A1 by pregnancy is negated by atazanavir UGT1A1 inhibition.

#### 1.4.7. Other Compounds with Dual AAI Activity

The principle governing the repurposing of other drugs for use against SARS-CoV-2 is the fact that some host cellular targets interfering with the viral growth cycle, such as kinases, are broadly shared in the mechanisms of several viral infections and other conditions such as cancer, making these compounds potentially interchangeable between different types of diseases. These compounds include, among others, the Janus kinase (JAK1) inhibitor Ruxolitinib [55], used for the purpose of controlling the cytokine storm and consequent ARDS observed in severely ill COVID-19 patients (NCT04362137 and NCT04377620, phase III clinical trials); plitidepsin, which blocks a human cell protein (eEF1A) that is required for SARS-CoV-2 infection [56]; and zotatifin, which emerged as an effective drug in reducing viral infectivity through eIF4A blockade, along with other compounds such as ternatin-4. Toremifene, carfilzomib, dactinomycin, and valrubicin are some other compounds that are potential candidates for repurposing towards the treatment of SARS-CoV-2 [57].

### 1.5. Computer-Aided Drug Design and Its Application to COVID-19

Drug discovery is a long-lasting and costly process and it takes around ten to fifteen years for a drug to reach the market [58]. Drug discovery begins with the identification of the appropriate drug target, lead discovery, and optimization of lead molecules and finishes with preclinical and clinical studies [59]. However, in most cases, the success rate of drugs through clinical trials is only 13%, usually due to a lack of optimal pharmacokinetic properties and toxicity [60].

**Figure 1:**
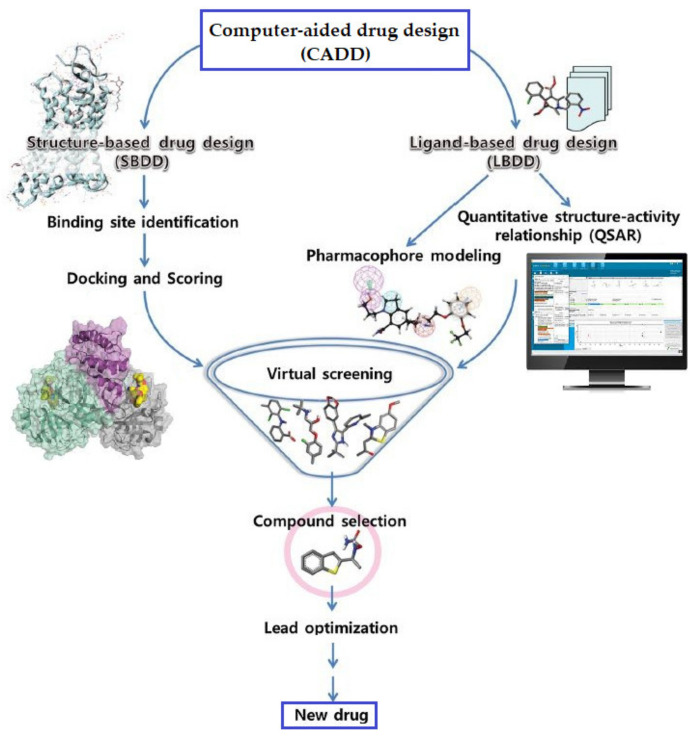
Drug discovery process and computer-aided drug design.

Nowadays, computer-aided drug discovery (CADD) techniques are widely used in preliminary studies by researchers and to accelerate the drug discovery and development process, while also reducing the costs and failures in clinical trials [61]. Rational drug design is a vital part of CADD that helps in the understanding of the binding affinity and molecular interactions between a target protein and a ligand, and it has been simplified by the development of supercomputers, parallel processing, and advanced programs and algorithms. Additionally, the current improvements in machine learning methods have significantly supported the analysis of pharmaceutical-related big data in the drug discovery process [62] (Figure 1).

In order to identify new inhibitors from chemical databases, different methods can be used, including pharmacophore modeling, quantitative structure–activity relationships (QSAR), molecular docking, quantum mechanics, and statistical methods. For the identification of lead molecules, two drug design approaches to computer-aided drug discovery can be used, the structure-based and ligand-based. The structure-based drug design approach depends on the three-dimensional structure of the receptor to understand the interactions between the receptor and ligand, while ligand based-drug design depends on the interaction of known ligands with the receptor [63]. Computer-aided drug design has been used successfully over the years [64] and could play a critical role in the discovery of new drug candidates against coronavirus disease 2019 (COVID-19).

As mentioned before, the novel coronavirus disease is caused by a new coronavirus known as severe acute respiratory syndrome coronavirus 2 (SARS-CoV-2), which has high nucleotide sequence similarity with severe acute respiratory syndrome coronavirus (SARS-CoV) and Middle East respiratory syndrome coronavirus (MERS-CoV). As with both of these viruses, the genome of SARS-CoV-2 encodes nonstructural and structural proteins that play a key role in the virus life cycle and can be used as promising targets for the design and development of novel SARS-CoV-2 agents with the help of CADD [65]. Moreover, with the availability of the complete genome sequence of SARS-CoV-2 and X-ray structures of the viral proteins, computer-aided drug design could be an important tool for the identification of novel SARS-CoV-2 agents.

#### 1.5.1. Structure-Based Drug Design

Structure-based drug design is supported by the extended accessibility of the three-dimensional structures and binding sites of various therapeutic target proteins. With this accessibility, structure-based drug design can help in the identification, at a molecular level, of lead molecules against various diseases. Structure-based drug design uses different methods, including molecular docking, structure-based virtual screening, and molecular dynamics. These methods have been used by many pharmaceutical industries and researchers and aided in the development of many commercial drugs, such as amprenavir, a protease inhibitor of the human immunodeficiency virus, developed using molecular docking and molecular dynamics simulations [66]; norfloxacin, a topoisomerase II and IV inhibitor [67]; an antituberculosis drug, isoniazid, discovered through structure-based virtual screening and pharmacophore modeling [68]; and flurbiprofen, targeting cyclooxygenase-2, a nonsteroidal anti-inflammatory drug against rheumatoid arthritis and osteoarthritis, discovered through molecular docking [69]. SBDD follows a series of steps including target structure preparation, identification of the ligand binding site, preparation of a compound library, molecular docking, molecular dynamic simulation, and calculation of the binding free energy.

For the preparation of the target structure, the continued development of structural elucidation techniques such as X-ray and NMR has recently increased the availability of protein structures in the Protein Data Bank (PDB). On the other hand, the identification of the ligand binding site requires specific docking studies, but it can also be found from the X-ray crystallographic structures of proteins co-crystallized with substrates or inhibitors [70]. If there is no structure available in PDB, the protein binding site can be predicted successfully by many software programs, such as DoGSite Scorer [71], DEPTH [72], MetaPocket [73], and others. In this stage, the tested compounds that are bulky and do not fit well within the binding pocket are excluded from the lead identification process. Furthermore, a large number of compounds can be selected from databases such as ZINC (230 million purchasable compounds) [74], PubChem (111 million compounds) [75], ChEMBL (>1.6 million compounds) [76], and DrugBank (14,528 drug molecules) [77]; then, molecular docking studies can be performed in drug-like compounds in order to identify possible drug candidates.

Molecular docking is a computational method that studies the interactions between a target receptor and a ligand at a molecular level and ranks the ligands using various scoring functions [78]. There is a wide variety molecular docking programs that can be used, such as AutoDock [79], AutoDock Vina [80], GOLD [81], FlexX [82], GLIDE [83], etc. Molecular docking can be divided into two categories: flexible-ligand search docking and flexible-protein docking. The flexible-ligand search docking method generally uses algorithms such as systematic, stochastic, and simulation methods [84], while flexible-protein docking uses Monte Carlo and molecular dynamic methods [85].

#### 1.5.2. Ligand-Based Drug Design

Ligand-based drug design is another technique used broadly in computer-aided drug design when the three-dimensional structure of the target protein is not available. Based on the fact that structural similarities correspond to similar biological activities, we can use a set of active compounds against a target protein in order to identify the physicochemical and structural characteristics that are responsible for the biological activity [86]. Some of the most used techniques in ligand-based drug design are pharmacophore modeling and quantitative structure–activity relationships.

#### 1.5.3. Computer-Aided Drug Design in COVID-19

Computer-aided drug design has been extensively used in the drug discovery process for the SARS-CoV-2 virus. Till now, only a few molecules, which are generally repurposed approved drugs, have been investigated in clinical trials.

With the absence of approved drugs and affective vaccines for COVID-19 and the accessibility of the complete genome sequence of SARS-CoV-2 [87] and structural elucidation of its proteins, the research for novel antiviral agents against COVID-19 disease has been rapidly pursued.

Researchers are swiftly working on designing and identifying inhibitors against all possible viral key protein targets of SARS-CoV-2, such as the structural proteins spike, envelope, membrane, and nucleocapsid, and nonstructural proteins such as the main protease, which is also known as 3C-like protease 3CLpro, papain-like protease, RNA-dependent RNA polymerase, nsp16 2-O-methyltransferase, nsp15 endoribonuclease, and nsp13 helicase. The structures of all these proteins can be used for structure-based virtual screening for the identification of specific inhibitors of the target proteins.

Computer-aided drug design has been successfully used in the drug discovery process. Selvaraj et al., performed homology modeling and molecular dynamics (MD) simulation and managed to solve the three-dimensional structure of SARS-CoV-2 guanine-N7 methyltransferase (nsp14). Moreover, based on molecular docking and simulation studies, they proposed five TCM database compounds (TCM 57025, TCM 3495, TCM 5376, TCM 20111, and TCM 31007) as potential antiviral phytochemicals and COVID-19 therapeutics [88].

Gao et al., reported the physicochemical properties and subcellular localization of the SARS-CoV-2 N protein and, using mass spectrometry analysis and flow cytometry, discovered its biological function. The revealed twelve phosphorylated sites and nine potential protein kinase sites in the SARS-CoV-2 N protein may serve as potential targets for drug discovery [89].

Elfiky used computational approaches such as homology modeling, molecular dynamics simulations, and molecular docking to target the SARS-CoV-2 RNA-dependent RNA polymerase enzyme and reported the effectiveness of sofosbuvir, ribavirin, galidesivir, remdesivir, favipiravir, cefuroxime, tenofovir, and hydroxychloroquine in binding to SARS-CoV-2 RNA-dependent RNA polymerase as candidate drugs for clinical trials [90].

Das et al., utilized a blind molecular docking approach to identify potential inhibitors of the SARS-CoV-2 main protease, by screening 33 molecules including natural products, as well as antiviral, antifungal, antinematode, and antiprotozoal agents. The highest inhibitory potency was found for rutin, a natural compound, followed by ritonavir (control drug), emetine (antiprotozoal), and indinavir (antiviral) [91].

Gurung et al., used a library of phytochemicals with reported antiviral activity for the identification of small inhibitors against the SARS-CoV-2 main protease (Mpro) using a molecular docking approach. They identified three antiviral phytochemicals, namely bonducellpin D, 5, 7-dimethoxyflavanone-4-O-β-d-glucopyranoside, and caesalmin B, as potential inhibitors of SARS-CoV-2 Mpro, SARS-CoV Mpro, and MERS-CoV Mpro [92]. In the same direction, using molecular docking studies, Joshi et al., identified natural molecules such as δ-viniferin, myricitrin, taiwanhomoflavone A, lactucopicrin 15-oxalate, nympholide A, afzelin, biorobin, and phyllaemblicin B as potential inhibitors of SARS-CoV-2 MPro [93]. Wahedi et al., with the aid of molecular docking and molecular dynamics simulation studies, identified piceatannol and resveratrol, from stilbenoid analogues, as important lead molecules to disrupt the formation of the SARS-CoV-2 and ACE-2 complex [94].

Khan et al., using molecular docking and molecular dynamics simulation studies, targeted chymotrypsin-like protease (3CLpro). The results showed that three FDA-approved drugs, namely remdesivir, saquinavir, and darunavir, and two natural compounds, namely flavone and coumarin derivatives, could act as promising inhibitors of the chymotrypsin-like protease enzyme [95]. Moreover, Carnosol, arjunglucoside-I, and rosmanol, as potent inhibitors of the SARS-CoV-2 Mpro enzyme, were identified by Umesh et al., by screening chemical species from Indian spices using molecular docking and molecular dynamics simulation studies [96].

Al-Khafaji et al., performed a covalent docking screening procedure joined with molecular dynamics simulation studies to identify molecules that can form a covalent bond with residue Cys145 within the binding pocket of the SARS-CoV-2 main protease and identified saquinavir, ritonavir, and remdesivir as the FDA-approved drugs that were the most promising inhibitors of SARS-CoV-2 Mpro [97]. Moreover, Peele et al., screened FDA-approved antiviral and antimalarial drugs through molecular docking studies and identified lopinavir and amodiaquine as promising inhibitors of the SARS-CoV-2 main protease [98]. Wang, using virtual docking, MD simulation, and binding free energy calculation, screened the majority of approved drugs and drug candidates in clinical trials and identified carfilzomib, eravacycline, valrubicin, and lopinavir as potential inhibitors of the SARS-CoV-2 main protease [99]. In this direction, Kandeel and Al-Nazami, using molecular modeling and a virtual screening approach, identified ribavirin and telbivudine as possible inhibitors of SARS-CoV-2 Mpro from a set of FDA-approved drugs [100]. FDA-approved antimicrobial drugs were also screened using a combined approach of molecular docking and molecular dynamics simulation by Mahanta et al., proposing, from the results, viomycin as a potential inhibitor of SARS-CoV-2 Mpro [101].

Beura and Chetti used pharmacophore modeling, molecular docking, binding free energy calculation, and ADME property analysis in order to study some chloroquine derivatives as SARS-CoV-2 Mpro inhibitors. From their study, it was found that molecule CQD15 is a promising inhibitor of the SARS-CoV-2 main protease, superior to chloroquine and hydroxychloroquine [102].

Kumar et al., using molecular docking studies, screened a hydroxyethylamine (HEA—pharmacophore derived from indinavir)-based library of chemical compounds and identified compound 16 as a promising inhibitor of SARS-CoV-2 3CLpro. Moreover, in MD simulation studies, this compound showed drug-like properties and stable binding within the binding pocket of SARS-CoV-2 3CLpro [103]. Likewise, Arun et al.,generated an E-pharmacophore hypothesis using the crystal structure of SARS-CoV-2 in complex with an imidazole carboxamide inhibitor. They used it to perform molecular docking and dynamic stimulation studies, which found that binifibrate and bamifylline drugs bind strongly within the enzyme active site pocket [104].

Amin et al., in order to perform the virtual screening of some inhouse chemicals, created a Monte Carlo optimization-based QSAR model. Among all screened compounds, thirteen showed good drug likeness scores in the SwissADME in silico study. Additional molecular docking studies revealed that these compounds interact in a favorable way with enzyme SARS-CoV-2 PLpro and thereby could be potential inhibitors of SARS-CoV-2 PLpro [105]. Similarly, Ghosh et al.,used the Monte Carlo optimization-based QSAR model to screen a library of nature product hits. The resulting active molecules were further analyzed from the aspects of fragment analysis, which revealed that novel potent SARS-CoV-2 Mpro inhibitors may be synthesized by joining fragments/features together or attaching them with other scaffolds [106].

The pharmacophore modeling approach plays an important role in the identification of lead molecules for drug discovery against COVID-19. A ligand-based pharmacophore model was generated by Law et al., using known antiviral drugs and was used to estimate the antiviral activity of twenty vanillin derivatives as SARS-CoV-2 Mpro inhibitors. Further results from the structure-based pharmacophore modeling approach suggested that twelve vanillin derivatives exhibited promising results as potent COVID-19 antiviral active compounds [107].

In the same way, Daoud et al., used the X-ray crystallographic structure of the COVID-19 main protease to construct a pharmacophore model and performed molecular docking studies in order to identify antiviral drugs as potential COVID-19 main protease inhibitors. Five FDA-approved antiviral drugs—lopinavir, remdesivir, ritonavir, saquinavir, and raltegravir—were successfully captured by the developed pharmacophore model and docked inside the binding site of COVID-19 Mpro. Docking studies revealed that these compounds exhibited specific binding interactions within the Mpro binding pocket that were comparable to those of the co-crystallized inhibitor (X77) [108].

Singh et al.,identified five compounds, viz., paritaprevir, glecaprevir, velpatasvir, remdesivir, and ribavirin, from a library of 1764 antiviral drugs against SARS-CoV-2 NSP12 (RNA polymerase), which exhibited high binding affinity with the drug target, performing docking-based virtual screening [109]. Finally, Ibrahim et al., combined molecular docking and molecular dynamics approaches to explore the potentialities of eighteen repurposed drugs in clinical development against SARS-CoV-2 Mpro. The results revealed that TMC-310911 and ritonavir could be promising drugs for the treatment of COVID-19 [110].

In addition, Petrou et al. [111] combined traditional medicinal chemistry, structural biology, and computational chemistry. They designed a series of new compounds that combine, in their structures, the minimum pharmacophores required to inhibit the main protease of SARS-CoV-2 Mpro, using molecular docking studies. The experimental data revealed that, among the fifteen compounds chosen, five compounds showed inhibitory activity with IC50 values within the range of 0.01–34.4 μΜ. It is noteworthy that these data provide evidence on the potential antiviral activity of these compounds against the main protease of SARS-CoV-2, to serve as potential candidates for COVID-19 therapeutics.

### 1.6. PBPK Modeling of Anti-COVID-19 Small Molecules

The repurposing of existing drugs that inhibit inflammatory pathways or similar enzymes of other viruses, and the development of synthetically novel agents, are viable strategies for the discovery of new anti-COVID-19 therapeutics. Whether one is referring to existing drugs that are repurposed to serve the goal of tackling a novel viral threat, or to new drugs under development, the capacity to predict drug concentration profiles in the tissue of interest represents a recurring challenge to accurate dosing. At the same time, the capability to predict drug–drug interactions (DDIs) is of paramount importance in complicated patients with multiple comorbidities. To this end, the advent of physiologically based pharmacokinetic (PBPK) modeling, a branch of quantitative systems pharmacology (QSP), is at the vanguard of modern pharmacology and has provided the industry and regulatory environment with powerful new methodologies that have reshaped drug development paradigms [112]. The main difference between classical descriptive (compartmental) and PBPK models lies in the distinction between system, drug, and trial data [113] (Table 2).

PBPK models are based on in vitro and in vivo correlation (IVIVC) procedures. Striving to be as mechanistic as possible in nature, they are based on the underlying anatomical, physiological, and biochemical characteristics of an organism [114]. In such models, the body is a multicompartment system, with every compartment representing a different organ, connected to other compartments by blood or lymph circulation, through a system of differential equations describing different physiological processes, such as blood flow, cardiac output, organ volumes, and glomerular filtration rate [115]. Such capability yields advantages to PBPK models that lead to better identification of the sources of PK variability, allowing extrapolation to different subpopulations [116].

In this context, precision medicine may be achieved in clinical therapeutics by connecting PBPK models with pharmacodynamic (PD) prediction models and their capacity for population simulation through the prediction of the effects of age, gender, comorbidities, genetic polymorphisms, and lifestyle factors such as smoking [114]. Finally, an advantage of such models is their ability to simulate the drug concentration profile at the site of action in the targeted organ or tissue, allowing the refinement of dosage schemes and the achievement of maximum safety and effectiveness profiles [117]. PBPK modeling may be especially valuable in the case of advanced formulations, such as nanoformulations, as the combination of knowledge about the structure and function of target organs with the physicochemical properties of the nanocarriers, the individual parameters of each patient, and the drug properties may create favorable conditions for individualized treatment [118]. Several platforms exist that utilize PBPK modeling approaches, including Simcyp (https://www.certara.com/software/simcyp-pbpk/, accessed on 1 May 2022), Gastroplus (https://www.simulations-plus.com/software/gastroplus/, accessed on 1 May 2022), and PKSim (http://www.systems-biology.com/products/PK-Sim.html, accessed on 1 May 2022), to name some of the most commonly used.

Conducting numerically robust clinical trials for the treatment of SARS-CoV-2 is challenging. In their work, Geerts, H. and P. van der Graaf suggest that the combination of PBPK modeling with other QSP approaches could provide a viable alternative to the continuation of clinical trials by creating virtual twin patients in order to study complex clinical datasets in a biologically and therapeutically relevant manner [119]. Importantly, the capability of combining PBPK models with pharmacodynamic (PD) and viral dynamic models provides the basis to develop more sophisticated predictive models [24]. The approach of creating individual pan-simulation models could permit the precise prediction in real time of the PK/PD profiles of therapeutics. This can be achieved by also exploiting bioinformatic data of individual patient signaling networks that are involved in PK/PD processes [118].

PBPK modeling approaches are especially useful in addressing urgent healthcare threats, as they contribute to: (a) individualizing the dosage schemes of currently used therapeutics to different subpopulations; (b) adjusting the dosage in order to develop appropriate concentration levels in specific tissues; (c) predicting the DDIs of different drugs used either as combination therapies or because of comorbidities; (d) identifying possible new therapeutics; and (e) calculating the appropriate dosage for repurposed drugs already in the market. The contribution of these simulation methodologies by effectively addressing scientific issues in model-informed drug development supports the productivity rates in the pharmaceutical sector, as well as the therapeutic efficacy and safety profiles in the clinical setting, especially for specific populations [120].

Pilla Reddy and colleagues used PBPK models to study changes in the PK parameters and DDI risks of different drugs that were repurposed for COVID-19 in different populations (e.g., geriatric), race groups, and physiologies (e.g., renal impairment) [121]. Using these models, they also predicted the exposure of epithelial lining fluid (ELF) for different drugs, a parameter that is relevant for patients with elevated cytokine levels. They found that in order to attain therapeutically relevant exposures for most drugs, no dose adjustments were warranted, except in the case of renal or hepatic impairment. Finally, by considering ELF exposure, they found that most drugs could achieve the target exposure, with the exception of hydroxychloroquine, azithromycin, atazanavir, and lopinavir–ritonavir.

**Table 2:** Application of PBPK in repurposing COVID-19 drugs.

**Drug**	**Description**	**Purpose of PBPK Model**	**Findings/Outcome**	**References**
Remdesivir	Extrapolate adult PBPK models to pediatric populations	Predict pediatric PK profile of remdesivir and metabolites in steady state	Predicted pediatric profiles	[23]
	Hybrid model with each tissue presented as two compartments	To predict remdesivir TN metabolite concentration in different tissues	Clinical dosing regimens successful in achieving desired TN concentrations	[24]
	Simulation of physiological properties and PK profiles	Examining DDI potential and properties in special populations	GS-5734 superior to remdesivir.Remdesivir shows no significant immunomodulatory activity	[25]
Ritonavir–Lopinavir	Models for Caucasian and Chinese populations	To examine the adequacy of current 400/100 mg BID dosing scheme in achieving adequate lung and plasma concentrations	Higher concentrations achieved in the Chinese population, but significant dose increase required to reach EC_50_ in both populations	[29]
Ritonavir–Nifedipine	Development and validation of PBPK models for ritonavir and nifedipine	Examine the DDI potential between ritonavir and nifedipine as they are frequently co-administered	Strong interaction that could lead to severe hypotension	[30]
Nitazoxanide	Develop a model for nitazoxanide	Calculate an optimal dosing scheme for repurposed drug nitazoxanide	Predicted optimal dosing schemes, providing rational basis for clinical trials	[38]
Chloroquine, Hydroxychloroquine, and Azithromycin	Utilizing PBPK with mechanistic lung model	Predict PK profiles in lungs as they are affected by changes in lung pH	Reduction in lung pH can lead to increased lung exposure with minimal plasma changes.Renal impairment increases local exposure	[42]
Chloroquine	Extrapolate adult PBPK models to pediatric populations	Calculate the optimal pediatric dose for different ages	Optimal dosing calculated to avoid suboptimal or toxic drug levels in children	[43]
Chloroquine	Create PBPK models for chloroquine using drug data extrapolated from animals	Sources to predict the concentration profiles of chloroquine in different tissues	Proposed optimized dosing regimens	[44]
Hydroxychloroquine	Create a PBPK model for hydroxychloroquine by focusing on drug absorption and disposition mechanisms	To support dosing design in specific populations (concomitant medications, age, organ impairment, pregnancy) to inform clinical trials	Proposed optimized dosing regimens	[45]
Atazanavir	Create PBPK model for atazanavir incorporating pre-absorptive and post-absorptive behavior	To identify the factors that contribute to the oral absorption of atazanavir	Post-absorptive factors more significant and thus formulation modification does not induce significant changes	[50]
	Evaluating population PBPK models	To predict the potential for DDIs in UGT1A1 in pregnant women	Induction of UGT1A1 by pregnancy was negated by atazanavir UGT1A1 inhibition	[51]

### 1.7. A Combined CADD/PBPK Approach

**Figure 2:**
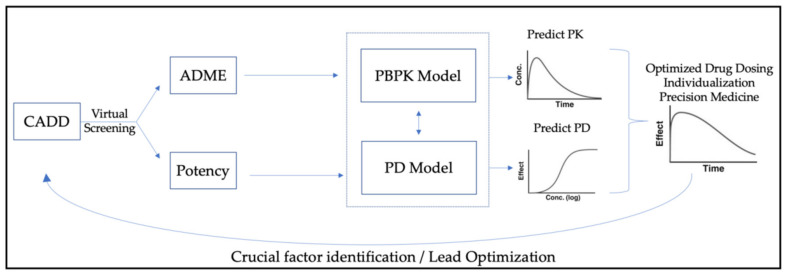
Combining PBPK and CADD methodological tools to accelerate the drug development process. PBPK modeling identifies sources of PK variability and predicts PK profiles in specific target organs and tissues. When combined with PD modeling, PBPK also generates predictive safety and efficacy profiles. The produced outcomes are utilized in a “reverse translation” approach to inform CADD. Iterative cycles of inputs and outputs may elucidate the optimal structure and lead to the best combination of PK, PD potency, as well as specific ADME tissue-targeted pharmacological properties.

To empower or strengthen drug development and productivity, both PBPK and CADD present useful methodological tools in the modern pharmaceutical era. While CADD can be used early on in preliminary studies to speed up the drug development process, PBPK modeling can provide useful insights both in identifying the sources of PK variability and in predicting the PK profiles in specific target organs or tissues. Furthermore, when coupled with PD modeling, PBPK can generate predictions regarding the safety and efficacy profiles. These computational approaches could be combined in series in order to gain earlier and cheaper information regarding new prospective molecules. At the same time, the produced outcomes can be utilized in a “reverse translation” approach to inform CADD. These cycles of inputs and outputs can elucidate the optimal structure that leads to the best combination of PK, PD potency, and also to achieve specific ADME properties upon creating tissue-targeted pharmacological moieties. This approach is graphically represented in Figure 2. A similar approach, termed “model-based target pharmacology assessment” (mTPA), has been utilized by GlaxoSmithKline [122]. Furthermore, the employment of machine learning (ML) and other artificial intelligence (AI) methodologies in such processes would further expand their predictive capacity [123].

## 2. Conclusions

The pathogenesis of COVID-19 involves direct viral infection and the inflammatory response to the pathogen and damaged host cells. Current standards of care for established infection include antiviral therapeutics (remdesivir, molnupiravir, and nirmatrelvir/ritonavir), anti-inflammatory agents (dexamethasone, baricitinib, and tocilizumab), and monoclonal antibodies binding to the spike protein of SARS-CoV-2 and preventing viral attachment to the human ACE2 receptor (bamlanivimab plus etesevimab, bebtelovimab, casirivimab plus imdevimab, and sotrovimab). Small molecules that possess dual antiviral and anti-inflammatory (AAI) activity may simplify therapy, prove to be more effective, prevent relapse, and reduce long-term COVID-19 complications. Dual AAI molecules may be especially helpful in resource-challenged countries, where access to new anti-inflammatory agents and to monoclonal antibodies may be limited. The discovery and development of dual-mechanism antiviral/anti-inflammatory small molecules may be accelerated by in silico structure-based and ligand-based computer-aided drug design using molecular docking and QSAR methods. Physiologically based pharmacokinetic (PBPK) modeling may be used to further optimize candidate AAI molecules and provide essential data for the accurate dosing of new therapeutic agents against COVID-19.

## Figures and Tables

**Table 1:** Mechanisms of action of antiviral agents.

Antiviral Agents with No Significant Anti-Inflammatory Activity		
Antiviral Agent	Structure	Mechanism of Action
**Remdesivir**	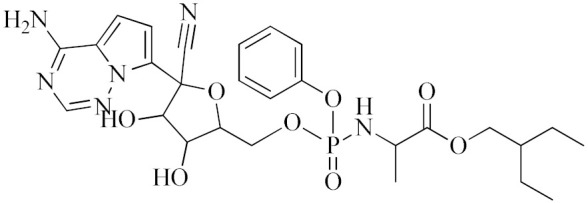	Competitive inhibitor of RNA-dependent RNA polymerase (RdRp)
**Lopinavir–Ritonavir**	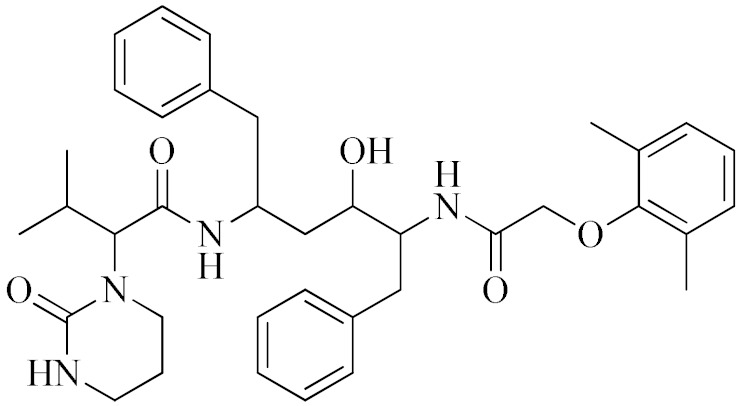	HIV-1 protease inhibitor used in combination with ritonavir to treat human immunodeficiency virus (HIV) infection.
**Molnupiravir**	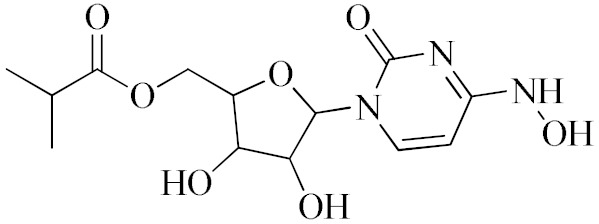	Mechanism of “error catastrophe”, by increasing the rate of mutation of the viral genome beyond a certain threshold.
**Nirmatrelvir–Ritonavir**	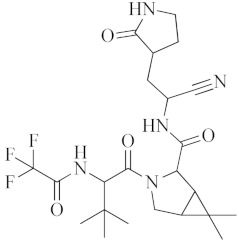	Inhibition of the main protease of SARS-CoV-2
**Dual-activity compounds as potential drug candidates against COVID-19**		
**Nitazoxanide**	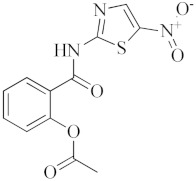	Inhibitor of several pro-inflammatory cytokines including IL-6, IL-8 and TNF- α in peripheral blood mononuclear cells.
**Chloroquine**	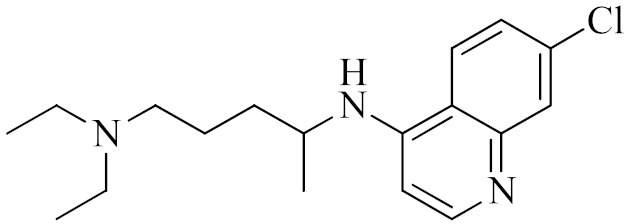	Inhibitor of heme polymerase in malarial trophozoites, as well as Toll-like receptors (TLRs).
**Hydroxychloroquine**	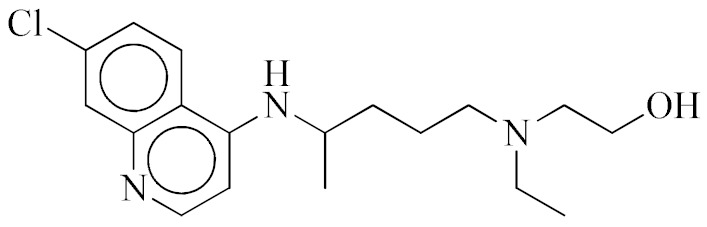	Inhibitor of Toll-like receptors, raising the pH in endosomes and preventing virus particles (such as SARS-CoV and SARS-CoV-2) from entering into the cell.
**Azithromycin**	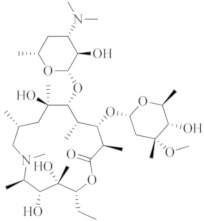	Macrolide antibiotic, stops bacterial protein synthesis by inhibiting the transpeptidation/translocation step of protein synthesis and by inhibiting the assembly of the 50S ribosomal subunit.
**Sabizabulin (Veru-111)**	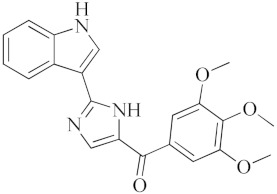	Targeting, binding to, and crosslinking the alpha and beta tubulin subunits of microtubules, as well as targeting the viral cytopathic pathway.
**Opaganib**	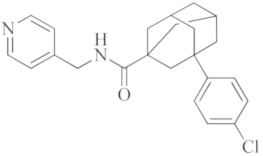	Inhibitor of sphingosine kinase-2.
**Selinexor**	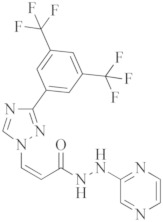	Inhibitor of nuclear transport (SINE) compound. Possesses anti-inflammatory activity by blocking expression of NF-κB-mediated cytokines, including TNFα, IL-1β, G-CSF and IL-6.
**Atazanavir**	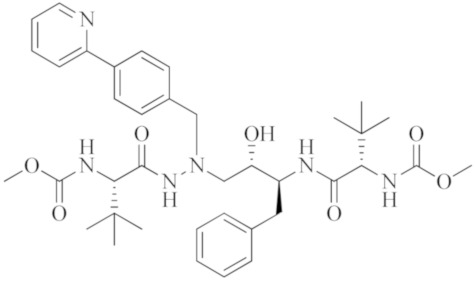	An anti-HIV drug, protease inhibitor.

## Data Availability

Not applicable.
